# Long-term outcome of multilayer flow modulator in aortic aneurysms

**DOI:** 10.2478/raon-2024-0021

**Published:** 2024-04-14

**Authors:** Karlo Pintaric, Lucka Boltezar, Nejc Umek, Dimitrij Kuhelj

**Affiliations:** Clinical Institute of Radiology, University Medical Center Ljubljana, Slovenia; Department of Radiology, Faculty of Medicine, University of Ljubljana, Slovenia; Department of Medical Oncology, Institute of Oncology Ljubljana, Slovenia; Faculty of Medicine, University of Ljubljana, Slovenia.; Institute of Anatomy, Faculty of Medicine, University of Ljubljana, Slovenia

**Keywords:** multilayer flow modulator, aortic aneurism, long-term follow up, volumetric measurements

## Abstract

**Background:**

This retrospective study investigated the efficacy of endovascular treatment with multilayer flow modulators (MFMs) for treating aortic aneurysms in high-risk patients unsuitable for conventional treatments.

**Patients and methods:**

Conducted from 2011 to 2019 at a single center, this retrospective observational study included 17 patients who underwent endovascular treatment with MFMs. These patients were selected based on their unsuitability for traditional surgical or endovascular procedures. The study involved meticulous pre-procedural planning, precise implantation of MFMs, and follow-up using CT angiography. The primary focus was on volumetric and flow volume changes in aneurysms, along with traditional diameter measurements. Moreover, the technical success and post-procedural complications were also registered.

**Results:**

The technical success rate was 100%, and 30-day procedural complication rate was 17.6%. Post-treatment assessments revealed that 11 out of 17 patients showed a decrease in flow volume within the aneurysm sac, indicative of a favorable hemodynamic response. The median decrease in flow volume was 12 ml, with a median relative decrease of 8%. However, there was no consistent reduction in aneurysm size; most aneurysms demonstrated a median increase in volume for 46 ml and median increase in diameter for 18 mm.

**Conclusions:**

While MFMs offer a potential alternative for high-risk aortic aneurysm patients, their effectiveness in preventing aneurysm expansion is limited. The results suggest that MFMs can provide a stable hemodynamic environment but do not reliably reduce aneurysm size. This underscores the need for ongoing vigilance and long-term monitoring in patients treated with this technology.

## Introduction

Endovascular management of aortic aneurysms with stent grafts has been possible for over 30 years. One of the challenges of the procedure is the preservation of flow in the aortic branches and the prevention of ischaemia. There have been many attempts to overcome this problem, including using fenestrated and branched stent grafts, as well as snorkel and chimney techniques.^1,2^ However, all these technical solutions are relatively complex and require appropriate anatomical conditions and highly skilled operators since the learning curves for the implantation of these devices are very prolonged.^3^ As an alternative, multilayer flow modulators (MFMs) have been developed. These are 3-layer stents designed to laminate the flow through the aneurysm so that it gradually becomes thrombotic without affecting the flow in the branches, simultaneously decreasing the peak stress on the aneurysm wall.^4^ This feature is particularly important in the aortic arch and thoracoabdominal region.

Most studies on MFM in the aorta have relied on measurements of aneurysm diameters, which is the most practical and time-efficient method. However, this approach does not provide comprehensive information on gradual thrombosis of the aneurysm and morphological changes in the aneurysm itself.^5,6,7,8^ Volumetric measurements and measurements of the flow volume through the aneurysm could provide additional information about the behaviour of aneurysms over time and, thus, about the efficiency of MFM stents. Accordingly, our study aimed to evaluate the safety and efficacy of MFMs implanted between 2011 and 2019 in a single centre cohort, using volumetric and flow volume assessments along with diameter measurements.

## Patients and methods

This retrospective observational study was approved by the Republic of Slovenia National Medical Ethics Committee (Permit No. 55/05/14) and was conducted in accordance with the World Medical Association Code of Ethics (Declaration of Helsinki). We analyzed a cohort of consecutive patients who underwent endovascular treatment for aortic aneurysms using MFMs (Cardiatis, Isnes, Belgium) at the University Clinical Center, Ljubljana, between March 2011 and October 2019. Our institution started using MFMs in 2011, and since then, we have implanted MFMs in 17 patients with aortic aneurysms.

Only patients unsuitable for open surgical treatment or other endovascular procedures were considered for endovascular treatment with MFM by a multidisciplinary board held for each patient. The exclusion criteria were rupture of aortic aneurysm, stenosis of branch arteries (arteries of head and neck, visceral and iliac arteries), occlusion of the aortoiliac segment, prior endovascular or surgical treatment of the same aneurysm, mycotic aneurysm, myeloproliferative blood disorders, known coagulopathies, and expected survival less than six months.^9^ For each patient, a consultation was held with Cardiatis (Isnes, Belgium) before the procedure to obtain their consent for MFM implantation.

Planning of the procedure was primarily performed by experienced interventional radiologists using computed tomographic (CT) angiography. Critical parameters assessed and measured prior to MFM implantation included the status of outgoing arteries (especially eventual stenoses), the largest diameter of the aneurysm, the diameter of the healthy vessel above and below the aneurysm, and the length of the aneurysm. Diagnostic images were shared with the MFM manufacturer and operator, who subsequently performed size of device determination, applying an oversizing of 10−15%. The total volume and flow volume of the aneurysms were estimated using the OsirX (Pixmeo, Geneve, Switzerland). These two parameters were also assessed in the last follow-up CT angiographies.

**FIGURE 1.**
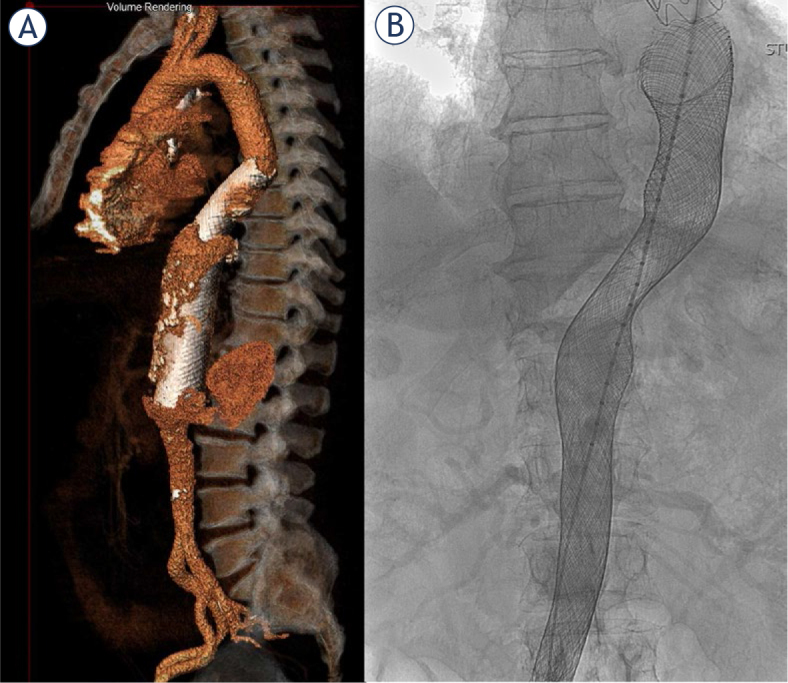
Volume rendering of implanted multilayer flow modulators (MFMs) **(A)** and fluoroscopic image of implanted MFM **(B)** in thoracoabdominal aorta.

All procedures were performed under general anesthesia. Prophylactic antibiotics were administered prior to the procedure, and 5000 IU of Heparin was administered during it. Bilateral access in the groin was achieved, and a large-bore sheath (22−24 Fr) was implanted at the site with larger iliac arteries through the common femoral artery to accommodate the MFM delivery system. The contralateral side was utilized to provide a diagnostic catheter, essential for precise MFM implantation. The MFM was carefully implanted using a slow release and push-pull technique to ensure adequate sealing of the affected aortic segment. In all patients, complete percutaneous hemostasis was successfully achieved using the ProStar XL/ProGlide systems (Abbott Laboratories, IL, USA). Following the procedure, all patients were prescribed lifelong treatment with acetylsalicylic acid and a three-month course of clopidogrel. Figure 1 shows volume rendering of aorta with implanted MFM and fluoroscopy image of implanted MFM in thoracoabdominal aorta.

The follow-up period in this study extended from the date of MFM implantation to the most recent CT angiography available in the hospital information system. Patient survival status, as of May 2023, was determined based on the cause of death listed in the National Registry.

For each patient, the technical success of the intervention, procedure-related complications, adherence to usage instructions, and mortality were documented. Technical success was defined as the accurate deployment of MFM at the targeted sites with the absence of any leaks at the attachment sites or the junctions of different device components.^5^

Serious adverse events such as cerebral stroke, renal ischemia, paraplegia, and aneurysm rupture were recorded. Complications attributed directly to the procedure were monitored at one month and twelve months after intervention. Furthermore, any potential reinterventions were noted, including the timing and cause for these additional procedures.

Additionally, the aneurysm diameters and volumetric data were calculated. Volumetric assessments of the aneurysms (total volume of the aneurysm sac and volume of aneurysm with retained flow) were performed prior to the implantation of the MFM and repeated in the last available CT angiography for each patient. The volumetric measurements were performed by Cardiatis (Isnes, Belgium). Incidences of branch occlusions, if any, were also reported.

### Statistical analysis

Statistical analysis was performed using GraphPad Prism 10 (GraphPad Software Inc., San Diego, CA, USA). Data are presented as median, interquartile range (IQR), range, frequency, and proportion. The comparison of numerical variables was performed using the Mann-Whitney test. Spearman’s correlations were used to test variable correlations. A *p*-value of 0.05 was considered statistically significant.

## Results

### Demographic data and clinical characteristics

During the study period, 17 patients were treated with MFM for aortic aneurysms, including 16 males and one female. The median age was 68 years (IQR: 66−78, range: 48–81 years). Four MFMs were implanted in aortic arch, six in thoracic aorta, one in thoracoabdominal aorta and two in abdominal aorta. The age of the patients at MFM implantation, the year of the first endovascular treatment, and the location of the placed MFM are shown in Table 1. All procedures were performed electively. The median duration of follow-up was 25 months, with an IQR of 13−58 months and an overall range of 7−76 months.

**TABLE 1.** Age of patients at procedure, year of procedure, and location of multilayer flow modulator (MFM)

**Patient**	**Age**	**Procedure year**	**Location of MFM**
1.	48	2012	Thoracic aorta
2.	79	2012	Thoracic aorta
3.	80	2012	Thoracic aorta
4.	73	2013	Aortic arch
5.	65	2013	Thoracic aorta
6.	67	2016	Aortic arch
7.	68	2016	Thoracic aorta
8.	63	2016	Abdominal aorta
9.	71	2016	Thoracic aorta
10.	80	2017	Abdominal aorta
11.	67	2017	Aortic arch
12.	65	2017	Aortic arch
13.	76	2018	Thoracoabdominal aorta
14.	75	2018	Thoracoabdominal aorta
15.	81	2018	Abdominal aorta
16.	66	2018	Abdominal aorta
17.	66	2019	Thoracoabdominal aorta

### Procedure and procedural complications

The procedure was performed according to the manufacturer’s instructions in 70.6% of patients. In other patients aneurysm was larger then 6.5 cm, which is not according to the manufacturer’s instruction of use.^8^ However, all procedures were performed with the consent of manufacturer. The technical success rate was 100%, and a total of 23 stents were implanted to treat all 17 patients.

The 30-day procedural complication rate was 17.6% (3/17). One patient required an additional stent graft after a few days to achieve an optimal proximal seal, as no additional MFM was available during the procedure. One patient was diagnosed with a type A dissection at CTA follow-up, which was attributed to the procedure itself, while one other was diagnosed with a dissection of the celiac trunk. Note, that the aforementioned type A dissection was stable on following CTAs, and cardiovascular surgeouns did not decide for surgical treatment. Additionally, one patient suffered a cerebral infarction due to a pre-existing stenosis of the left carotid artery and brachiocephalic trunk (however, poor adherence to antiplatelet medication was noted in this patient). None of the patients required open surgery due to these complications, and none developed paraplegia, end-organ failure, or aneurysm rupture.

### Mortality

The intraoperative and 30-day mortality rates were both 0%. After 12 months, the aneurysm-related mortality rate was 5.9% (1 in 17 patients). As of May 2023, 5 of the 17 (29.4%) patients were still alive, while 12 (71.6%) had died. Three of these deaths were due to aneurysm ruptures that occurred 9 months, 40 months, and 51 months after MFM implantation. The remaining deaths were attributed to other causes, including head and neck cancer, pneumonia, myocardial infarction, and suicide.

### Aortic branch occlusions

Aortic branch occlusions following the MFM implantation occurred in 5 patients (29.4%) – one patient developed chronic renal failure due to stenosis of the left renal artery (which did not require haemodialysis). Vascular occlusion in the other patients included stenosis of the superior mesenteric artery, left subclavian artery, superior mesenteric artery and the celiac trunk (with robust collaterals from the inferior mesenteric artery), and the left carotid artery and the brachiocephalic trunk.

### Reinterventions

Reintervention was required in 7 patients (41.2%) as shown in Table 2. Most reinterventions were performed in the early years of our practice due to type 1 leaks.

**TABLE 2.** Time and cause of reinterventions after multilayer flow modulator (MFM) implantation

**Patient number**	**Time after MFM implantation (months)**	**Cause of reintervention; reintervention undertaken**
1.	25	Endoleak type I and enlargement of the aneurysm; implantation of another MFM
2.	3	Insufficient proximal seal and collateral flow, resulting in enlargement of the aneurysm; implantation of another MFM
3.	1	Insufficient proximal seal; implantation of another MFM
4.	8	Enlargement of aneurysm and partial stenosis of subclavian artery; implantation of another MFM into previous MFM
6.	13	Stenosis of brachiocephalic truncus; implantation of a stent, which resulted in a stroke
9.	9	Migration of the MFM; implantation of another MFM
10.	74	Displacement of MFM and stent graft; implantation of another stent graft

### Changes in aneurysm size after MFM implantation

The median aneurysm volume before MFM implantation was 309 ml (IQR: 223−452 ml, range 32−856 ml) with a median diameter of 58 mm (IQR: 51−68 mm, range 26−96 mm). At the last follow−up, the median aneurysm volume was 355 ml (IQR: 237−608 ml, range: 62−881 ml), while the median diameter was 76 mm (IQR: 57−92 mm, range 27−103 mm).

Based on volume measurements, five patients (29.4%) experienced shrinkage of the aneurysm at the last follow-up, two (11.8%) experienced no volume change over time, and ten (58.5%) experienced an enlargement of the aneurysm during the observation period. In latter patients, the median enlargement of the sac was 96 ml (IQR: −15−117 ml, range −84−718 ml) in volume and 15 mm (IQR: 3−27 mm, range: −7−49 mm) in diameter. Nine of these patients had an enlargement of the aneurysm for more than 10% of the initial volume.

Per the measurements of the maximum diameter, fifteen patients (88.2%) experienced an increase in the diameter of the aneurysm, one patient experienced shrinkage, and in one patient, the diameter remained stable during the follow-up period. Patient-specific measurements are presented in Table 3.

**TABLE 3.** Aneurysm size before and after multilayer flow modulator (MFM) implantation

**Patient number**	**Aneurysm volume (ml)**		**Aneurysm diameter (mm)**		**Difference between the last follow-up and before the MFM implantation**		**Duration of follow-up (months)**

	**Before MFM implantation**	**At last follow-up**	**Before MFM implantation**	**At last follow-up**	**Volume (ml [%])**	**Diameter (mm [%])**	
1.	345	854	65	107	509 [147]	42 [64]	76
2.	888	920	122	130	32 [4]	8 [7]	9
3.	309	943	70	110	634 [205]	40 [57]	50
4.	255	355	57	93	100 [39]	36 [63]	69
5.	75	62	26	27	−13 [−17]	1 [4]	14
6.	197	330	44	76	133 [68]	32 [73]	49
7.	343	549	65	85	206 [60]	20 [31]	28
8.	311	266	48	68	−45 [−14]	20 [42]	3
9.	575	667	76	81	92 [16]	5 [7]	14
10.	539	530	97	90	−9 [−2]	−7 [−7]	40
11.	32	62	34	50	30 [94]	16 [47]	48
12.	411	411	54	55	0 [0]	1 [2]	33
13.	493	409	53	57	−84 [−17]	4 [8]	24
14.	262	332	60	75	70 [27]	15 [25]	23
15.	307	223	59	81	−84 [−27]	22 [37]	18
16.	130	113	58	58	−17 [−13]	0 [0]	19
17.	250	250	54	60	0 [0]	6 [11]	7

There were no significant correlations between the aneurysm volume before MFM implantation and absolute or relative change in the aneurysm volume after MFM implantation (*p* = 0.9167 and *p* = 0.4473, respectively). We only noted a significant positive correlation between the aneurysm volume before MFM implantation and the aneurysm volume at the last follow-up after MFM implantation (ϱ = 0.71, *p* = 0.0013) and between aneurysm volume and maximal diameter (ϱ = 0.86, R^2^ = 0.74, *p* = 0.0001).

There was a significant positive correlation between follow-up duration and both absolute and relative changes in aneurysm volume and diameter (absolute volume: ϱ = 0.62, *p* = 0.0084, relative volume: ϱ = 0.70, *p* = 0.0017, absolute diameter: ϱ = 0.65, *p* = 0.0046, relative diameter: ϱ = 0.66, *p* = 0.0040). Therefore, we normalized absolute and relative changes in aneurysm size to follow-up duration; however, again, there were no significant correlations between initial aneurysm size and normalized absolute and relative changes in aneurysm size after MFM implantation. Moreover, there were no significant correlations between initial aneurysm size or change in aneurysm size and patient age.

Three patients (17.6%) had complete occlusion of the aneurysm sac with a thrombus around the implanted MFM, twelve patients (70.6%) still had partial flow in the aneurysm sac (four of them, however, with only minimal presence of contrast medium on CT angiography), and two patients (11.8%) had a completely non-occluded aneurysm sac with flow still present in the aneurysm sac.

### Changes in aneurysm flow volume after MFM implantation

Six patients had an increase in flow volume (35.3%), and 11 patients had a decrease in flow volume (64.7%) at the last follow-up, which is considered a favourable haemodynamic outcome. The median volume before MFM implantation was 183 ml (IQR: 159−262 ml, range: 18−468 ml), and the median flow volume at the last follow-up was 168 ml (IQR: 125−268 ml, range: 6−555 ml). The median change in flow volume was −12 ml (IQR: −36−31 ml, range: −94−124 ml), and the median relative decrease in flow volume was 8% (IQR: −19%−15%, range: −64%−49%). We found no significant correlation between the patient’s age, duration of follow-up, or initial size of the aneurysm and the change in absolute or relative flow volume. There was also no significant correlation between absolute or relative change in aneurysm volume or diameter and absolute or relative flow volume.

## Discussion

We studied consecutive patients treated with MFM stents for aortic aneurysms between 2011 and 2019 in our centre. We found that flow volume decreased in 11 out of 17 patients after MFM implantation, which is considered a favourable hemodynamic response; however, this did not correlate with a decrease in aneurysm sac size. In 15 out of 17 patients, the aneurysm sac increased in diameter, which was also accompanied by an increase in volume in 10 out of 17 patients.

Our institution was one of the first centers to perform treatment with MFM stents for aortic aneurysms. This study, therefore, has one of the longest follow-up periods currently published in the literature. The literature review by Pinto *et al*. concluded that the implantation of MFMs is safe with few complications, although no randomized studies were available.^9^ Most of the studies published to date included a few patients, usually less than 30, with a relatively short follow-up period of no more than 12 months.^5,6,7,8,10,11,12,13,14,15^ A larger study with 103 patients was published by Sultan *et al*. in 2014 but with a short median follow-up of only six months.^15^ There are two studies that report a median follow-up time of 22 months: one from Ireland with 14 patients included^11^ and the other from Italy with only 8 patients included.^13^

The literature mostly reports measurements of the aneurysm maximal diameters.^5,6,7,8,10,11,12,13,14,15^ To our knowledge, our study is the first to evaluate volumetric measurements before MFM implantation and at the follow-up over a longer period. Two studies by Sultan *et al*., which included patients with first-generation MFMs, performed volumetric evaluation of aneurysm sacs over several months.^14,15^ The first from 2013 reported an overall mean increase in sac volume of 3.3% in 55 patients^14^, while the second study with 103 patients in 2014 reported an overall mean increase in sac volume of 5.1% and a mean volume change of 63 ml 12 months after implantation.^15^ In our series, the mean volume increase was 33.4% and 91 ml, which is significantly more than noted by Sultan *et al*. This could be due to the longer observation period in our study, which is also supported by a significant positive correlation between the follow-up period and the change in volume and diameter of the aneurysm sac.

The proportion of aneurysm sac expansion in our study is consistent with the findings of Lowe *et al*.^11^, who conducted a prospective study on patients treated with MFMs, characterized by a mean patient age of 74 years and a follow-up period of 22 months. Their findings revealed that none of the aneurysms demonstrated shrinkage. They reported a one-year all-cause survival rate of 79%, which dropped to 50% at two years. Remarkably, only two patients exhibited stable aneurysm sac diameters, with all others experiencing aneurysm sac volume expansion. While their conclusion does not support the continued use of MFMs, it is important to note that their study, conducted from 2011 to 2014, included only patients fitted with the first generation of the device. On the contrary, Vaislic *et al*. reported a very high proportion of stable aneurysm sac size (90%); however, the observational period was only 12 months.^16^

We found a strong correlation between aneurysm sac enlargement and follow-up period, which suggests that MFM implantation is probably not a lifelong stable solution as often advertised by the industry. Most of the aneurysm sacs became completely or partially obliterated by thrombus, which is consistent with the manufacturer’s caution. However, despite that, in almost two-thirds of patients, the flow volume decreased, and the aneurysm sac size increased both in volume and diameter. We also noticed a strong positive correlation between aneurysm volume and diameter change, suggesting that diameter measurements are probably sufficient for assessing aneurysm size at follow-up CT angiographies.

We observed that 41% of patients required reintervention, a rate lower than the approximately 77% reported by Ibrahim *et al*. in 2018.^8^ This discrepancy may stem from the urgency of treatments in the latter study, where most patients underwent emergency procedures. The selection of devices and implantation techniques in emergency scenarios differs markedly from those in elective procedures. Additionally, a notable proportion of reinterventions in our series was attributed to technical challenges associated with the first-generation MFMs, suggesting that device technology advancements may influence the need for subsequent interventions.

Existing literature suggests that aneurysm-related survival rates 18 months post-implantation of MFMs can be as low as 25.6% when these devices are used with deviations from the prescribed instructions.^15,17^ However, our data indicates more favourable long-term survival outcomes, despite 29.4% of the procedures in our study deviating from the recommended usage guidelines. A significant factor contributing to these improved outcomes is likely the elective setting in which our procedures were conducted, suggesting that the context of the procedure may play an important role in patient survival post-implantation.

In our study, we observed three deaths attributed to aneurysm rupture: one occurring 9 months, another after 40 months, and the third nearly 6 years post-MFM implantation. Although the aneurysm-related mortality was relatively low, the overall mortality rate was notably high. This may be partly attributed to the inclusion of patients unsuitable for surgical intervention, often due to their suboptimal general health conditions. Notably, the instance of an aneurysm rupture 6 years after MFM implantation underscores the importance of prolonged follow-up in these patients, highlighting the need for ongoing monitoring even years after the initial treatment.

The primary limitation of our study, as with many others focusing on MFMs, is the relatively small patient sample size. However, our research holds distinct value due to its nature as a consecutive series conducted by a consistently trained team in a single centre. Furthermore, the extensive duration of our follow-up and the employment of volumetric measurements offer a significant contribution to the existing body of knowledge in the field of intravascular treatments.

## Conclusions

MFMs present a viable treatment alternative for high-risk patients who are unsuitable for surgery and stent grafts. However, long-term real-life data show that while MFMs may not be as effective in preventing aneurysm expansion as originally thought, they can still provide a relatively stable haemodynamic solution over a prolonged period. Our study found a correlation between the duration of follow-up and the increase in aneurysm sac size. Although there are often no alternative treatment options for these patients and MFM insertion generally carries a low risk of periprocedural and long-term complications, careful, lifelong follow-up is essential to recognize early signs of deterioration and intervene appropriately.
